# Induction of germ cell-like cells from deleted in azoospermia-like enhanced *green fluorescent protein* gene knock-in chicken somatic cells via transgenic expression of pluripotency and germ cell-specific transcription factors

**DOI:** 10.5713/ab.25.0233

**Published:** 2025-08-12

**Authors:** Bo Ram Lee, Hyeon Yang, Sun Keun Jung, Sung June Byun, Tae Sub Park

**Affiliations:** 1 Animal Biotechnology and Genomics Division, National Institute of Animal Science, Rural Development Administration, Wanju, Korea; 2 Poultry Research Center, National Institute of Animal Science, Rural Development Administration, Pyeongchang, Korea; 3 Graduate School of International Agricultural Technology and Institute of Green-Bio Science and Technology, Seoul National University, Pyeongchang, Korea

**Keywords:** Chicken, Deleted in Azoospermia-like, Genome Editing, Germness, Reporter System

## Abstract

**Objective:**

Germ cell identity is regulated by the coordinated action of multiple key transcription factors during embryonic development, which includes the induction and control of germ-line-specific gene expression. The expression of *DEAD-box helicase 4* (*DDX4*) and *deleted in azoospermia-like* (*DAZL*) genes in chickens plays a pivotal role in germplasm formation and the specification of germ cell lineage from a totipotent genome. This study aimed to investigate the regulatory mechanisms underlying germ cell fate determination.

**Methods:**

Large-scale gene expression profiling was conducted to screen and select critical transcription factors. This analysis identified differentially expressed genes in chicken primordial germ cells (PGCs), comprising 1,020 transcription factors. Additionally, we generated a chicken DF1 cell line featuring an enhanced green fluorescent protein (eGFP) reporter precisely knocked into the transcriptional start site of the *DAZL* gene using the CRISPR-Cas9 system, enabling real-time monitoring of DAZL expression during reprogramming.

**Results:**

Through analysis of transcription factor binding sites within approximately 10 kb upstream regions of DDX4 and DAZL, resulting in the selection of 10 candidate transcription factors for germ cell induction. Subsequently, the ten transcription factors identified as regulators of germ cell identity were transduced into the DAZL-knock-in eGFP DF1 cells. This approach led to the successful induction of eGFP-expressing cells *in vitro*, driven by the endogenous DAZL promoter. We conducted further characterization of these cells to confirm their germ cell-specific properties.

**Conclusion:**

Our findings offer new insights into the transcriptional regulation of chicken germ cells by identifying key factors that activate DAZL expression. These results indicated valuable opportunities for advancing germ cell induction from somatic cells, with potential applications of *in vitro* models for studying germ cell-specific gene regulatory pathways in avian species.

## INTRODUCTION

Germ cell identity is regulated by the coordinated expression of germline-specific genes, including transcription factors (TFs) and RNA-binding proteins (RBPs) [1,2]. During early embryogenesis, primordial germ cells (PGCs) develop through specialized genetic programs that distinguish them from somatic cells. These programs are crucial for the proper specification, proliferation, migration, and differentiation of germ cells, ultimately leading to the formation of functional gametes [3,4]. However, the mechanisms of germline specification in avian species, such as chickens, differ significantly from those in mammals and remains controversial [5,6].

To date, two primary modes of PGC specification have been well characterized. In the epigenesis mode, typical of mammals, PGCs arise later in embryogenesis from pluripotent progenitors that are directed to become germ cells by external signals [7]. The preformation mode, observed in species such as zebrafish, fruit flies, frogs, and birds, involves PGC specification through specialized maternal cytoplasm [8]. Given their evolutionary position and distinct characteristics, chickens display both similarities to and differences from these two general modes of PGC specification, suggesting a blend of common and unique processes. Generally, PGC specification depends on mechanisms that suppress the expression of somatic genes through transcriptional inactivation and genome-wide chromatin remodelling. Following this, PGCs initiate the transcription of germline-specific genes [9].

Germ cell-specific genes, including TFs and RBPs, are crucial in regulating germ cell fate and development [10]. Recent studies have aimed to uncover the gene regulatory networks that govern germ cell fate in avian species. For instance, Lee et al identified the *deleted in azoospermia-like* (*DAZL*) gene as a key marker for monitoring germplasm formation and PGC lineage specification in chickens [6]. Furthermore, chicken NANOG knocked-in reporter DF1 cells were successfully created to track the induction of pluripotency during the generation of chicken induced pluripotent stem cells (iPSCs) [11]. Lu et al also demonstrated that chicken iPSCs can differentiate into germ cells and express several PGC marker genes [12].

In mammals, *in vitro* gametogenesis has successfully used embryonic stem cells (ESCs) and iPSCs to study PGC specification and is considered a valuable tool in the field of reproductive medicine as well as reproduction [13,14]. However, compared to mammals, our understanding of PGC specification and underlying molecular mechanisms including key factors and regulatory mechanisms controlling germ-line properties and stemness is still limited, despite the potential of chicken PGCs for genetic research and transgenesis [15–18]. Therefore, a precise reporter system to trace germplasm formation and PGC lineage specification is essential for studying these species-specific differences in chicken PGCs. In this study, we selected the key TFs involved in induction of germ cells and investigated their characteristics *in vitro* using DAZL knocked-in enhanced green fluorescent protein (eGFP) reporter DF1 cells generated through genome editing technology.

## MATERIALS AND METHODS

### Culture of chicken DF1 and primordial germ cells

Chicken DF1 fibroblast cells (CRL-12203; ATCC) were cultured and regularly subcultured in Dulbecco’s modified Eagle’s medium (DMEM; Invitrogen) supplemented with 10% foetal bovine serum (FBS; Hyclone) and a 1× antibiotic-antimycotic solution. The cells were incubated under conditions of 37°C, 5% CO_2_, and 60%–70% relative humidity. For PGCs, the culture medium used was knockout DMEM (Gibco) supplemented with 20% FBS, 2% chicken serum (Sigma-Aldrich), 1× nucleosides (Millipore), 2 mM L-glutamine (Gibco), 1× non-essential amino acids (Gibco), β-mercaptoethanol (Gibco), 1 mM sodium pyruvate (Gibco), 1× antibiotic-antimycotic (Gibco), and 10 ng/mL of human basic fibroblast growth factor (bFGF; Koma Biotech).

### Search and identification of differential expressed genes from large scale gene expression

We employed the microarray-based NCBI GEO dataset (GSE15830) and the whole-transcriptome sequencing (WTS)-based NCBI GEO dataset (GSE174603) to identify key genes associated with stemness and germ cell identity, particularly focusing on TFs expressed in stage X embryos and chicken PGCs compared to somatic cells such as gonadal stromal cells (GSCs) and chicken embryonic fibroblast cells (CEFs) [19,20]. Notably, differentially expressed genes (DEGs) identified through microarray analysis were significantly upregulated in chicken E6.5 gonadal PGCs compared to GSCs in the GEO dataset, as determined by Welch’s t-test (p-value<0.05) and a fold change threshold (FC>4.0). Additionally, differential gene expression analysis for WTS data was conducted using edgeR version 3.40.2, with raw counts serving as the input [21]. For quality control, genes that had non-zero counts in all replicates of at least one group were retained. The dataset was then normalized using the trimmed mean of M-value method to account for variations in library size across samples. The statistical significance of DEGs was assessed using the edgeR exact Test, with fold changes and p-values being extracted from the test results. To control the false discovery rate, all p-values were adjusted using the Benjamini-Hochberg method. The significant genes were filtered based on the following criteria: |fold change|≥2 and raw p-value<0.05. Significant genes were then hierarchically clustered using normalized values (distance metric = Euclidean distance; linkage method = complete). In gProfiler, raw p-values were calculated using a one-sided hypergeometric test and corrected using the Benjamini-Hochberg method. All data analyses and visualizations of DEGs were performed using R version 4.2.2 (www.r-project.org).

### Prediction of putative transcriptional binding sites using *in silico* sequence analysis

The predicted mRNA sequences of germplasm-associated genes, including *DDX4* and *DAZL*, were analysed using the University of California Santa Cruz BLAT program to identify their promoter regions. Approximately 10 kb of sequences upstream of these germplasm genes were then extracted in FASTA format and assessed for TF binding sites using the Genomatrix MatInspector software tool [22]. The analysis focused on TFs that were significantly expressed in chicken PGCs relative to stage X embryos and somatic cells such as GSCs and CEFs. TFs with at least one binding site within the promoter regions of these germplasm genes were selected for further overexpression studies.

### Generation of the CRISPR/Cas9-mediated deleted in azoospermia-like knocked-in reporter cell line

The DAZL gRNA and the eGFP-2A-puroR donor template, along with a Cas9 nuclease expression plasmid, were synthesized by Bionics and co-transfected using Lipofectamine 3000 reagent (Invitrogen). In brief, Cas9-GFP plasmids co-expressing DAZL gRNA and eGFP-2A-puroR donor templates were mixed with a solution containing 7.5 μL of Lipofectamine 3000 reagent, diluted in 250 μL of OPTI-MEM at room temperature. After a 5-minute incubation, the mixtures were combined and incubated for an additional 5 minutes. The mixture was then gently pipetted and applied to cultured chicken DF1 cells. One day post-lipofection, GFP-expressing cells were isolated using a FACS Aria III cell sorter (Becton Dickinson). Following cell harvest, the chicken DF1 cells were resuspended in phosphate-buffered saline (PBS) containing 0.1% bovine serum albumin (BSA) and filtered through a 40 μm cell strainer (BD Falcon; Becton Dickinson) for FACS separation. After sorting, the cells were cultured again in complete DMEM. To establish DAZL knocked-in eGFP DF1 cells, individual cells were isolated and cultured. Subsequently, single-cell-derived DAZL knocked-in eGFP DF1 sublines were identified through genomic polymerase chain reaction (PCR) analysis and sequencing using chDAZL KI primers (chDAZL KI F: 5′- tcc cct ttc cct ttt cct aa -3′, chDAZL KI R: 5′- gca gat acc gtg cgt aaa aa -3′) and of them, knockout of a single allele of DAZL was finally selected and used in this study.

### *In vitro* transfection

The coding sequences for chicken *NANOG* (NM_001146142), *POUV* (NM_001110178), *SOX2* (NM_205188), *LIN28A* (NM_001031774), *Blimp1* (XM_004940353), *TFAP2C* (XM_ 417497), *PRDM14* (XM_025147991), *HNF4A* (NM_ 001030855), *OTX2* (NM_204520), and *CDX2* (NM_204311) were synthesized and inserted downstream of the CMV early enhancer/chicken β-actin (CAG) promoter region in their respective expression vectors. Chicken DAZL knocked-in reporter DF1 cells were harvested following treatment with 0.05% (v/v) trypsin containing 0.53 mM EDTA and electroporated with each of the ten expression vectors using the Basic Fibroblast Nucleofector kit (Lonza VPI-1002) according to the manufacturer’s protocol. In brief, each vector was combined with 80 μL of basic nucleofector solution and 16 μL of supplement 1, and the mixture was carefully transferred into a Nucleocuvette Vessel for electroporation using the T-023 program (Lonza). The transfected cells were then seeded in a six-well plate containing DMEM (Invitrogen) with 10% FBS and 1× antibiotic-antimycotic solution. Two days post-transfection, the medium was removed, and the cells were replated onto a Matrigel-coated six-well plate. Three days after transfection, colonies were transferred to a Matrigel-coated six-well plate with mTeSR1 medium (Stem Cell Technology), and the medium was refreshed every two days. The cells were monitored for eGFP expression, and 21 days post-transfection, the culture medium was switched to PGC culture medium.

### Immunocytochemistry and flow cytometry analysis

The cells were analyzed via immunocytochemistry using the following primary antibodies to characterize DAZL promoter-driven eGFP-expressing cells: CVH (ab13840, rabbit, 1:100) and SSEA-1 (Thermo Scientific 41-1200, mouse, 1:200). Cells were fixed on glass slides in 1× PBS containing 4% paraformaldehyde for 10 minutes at room temperature. After two washes with 1× PBS, the cells were blocked with 1% BSA in 1× PBS with 0.1% Triton X-100 for 30 minutes at room temperature. They were then incubated overnight at 4°C with the primary antibody, followed by a 45-minute incubation with a fluorochrome-conjugated secondary antibody at room temperature. The cells were subsequently stained with 300 nM 4’ 6-diamidino-2-phenylindole (DAPI; Invitrogen) for nuclear visualization for 5 minutes, followed by multiple rinses with 1× PBS. Images were captured using either a Nikon AX confocal microscope (Nikon) or a fluorescence microscope (Leica Microsystems). For flow cytometry analysis, cells were harvested from 12-well plates by washing with 1× PBS and treating with Accutase (Invitrogen) for 3–5 minutes. After two washes with 1× PBS containing 0.75% BSA (Sigma-Aldrich), the cells were analyzed using a BD FACS Calibur flow cytometer (BD Biosciences) and FlowJo (v5.7.2) software (Tree Star).

### Quantitative reverse transcription polymerase chain reaction

Total RNA from the prepared samples was extracted using TRIzol reagent (Life Technologies), and quantitative reverse transcription polymerase chain reaction (RT-PCR) was conducted to verify whether expression of *DAZL* and *DDX4* is TF-mediated and further evaluate the expression of endogenous pluripotency markers and germplasm markers during induction process, as outlined in Tables 1, 2, respectively. The PCR reaction mixture was composed of 2 μL of 10 pmol each of forward and reverse primers, 7 μL of nuclease-free water, 10 μL of SYBR Green qPCR Master Mix, and 1 μL of cDNA, resulting in a final volume of 20 μL. Gene expression levels were quantified using the StepOnePlus Real-Time PCR System (Applied Biosystems) with GAPDH as the housekeeping gene for normalization, applying the 2-^ΔΔ^Ct method to determine relative gene expression.

### Statistical analysis

All data are presented as the mean±standard error of the mean (SEM) from three independent experiments. Statistical analysis to compare differences between experimental and control groups was performed using the general linear model (PROC-GLM) function in the SAS software. A p-value of 0.05 or less was considered statistically significant (* p<0.05, ** p< 0.01, and *** p<0.001).

## RESULTS

### Identification of the key transcription factors for inducing germplasm gene expression

**Figure 1:**
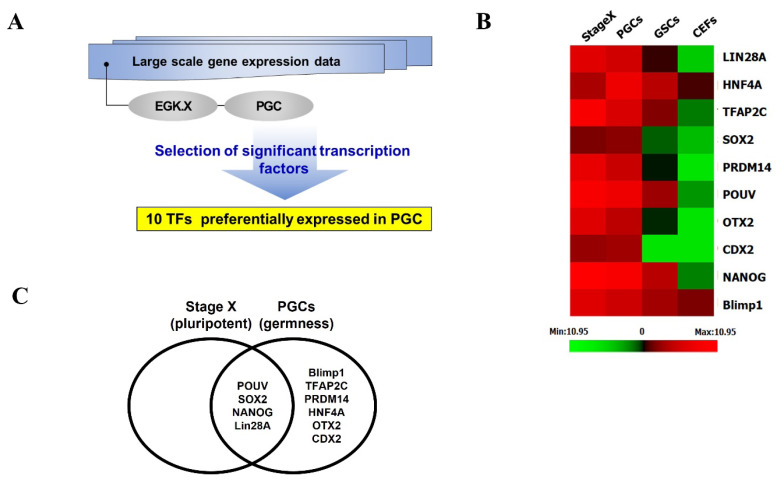
Screening of pluripotency- and germ cell identity-related genes. (A) The screening process of the key transcription factors (TFs) for the induction of chicken pluripotent and germplasm gene expression through the analysis of large-scale gene expression data. (B) Heatmap of differentially expressed genes (DEGs) between pluripotent cells and germ cells. (C) The 10-selected TFs in chicken germ cells. PGC, primordial germ cell; GSC, gonadal stromal cell; CEF, chicken embryonic fibroblast cell.

**Figure 2:**
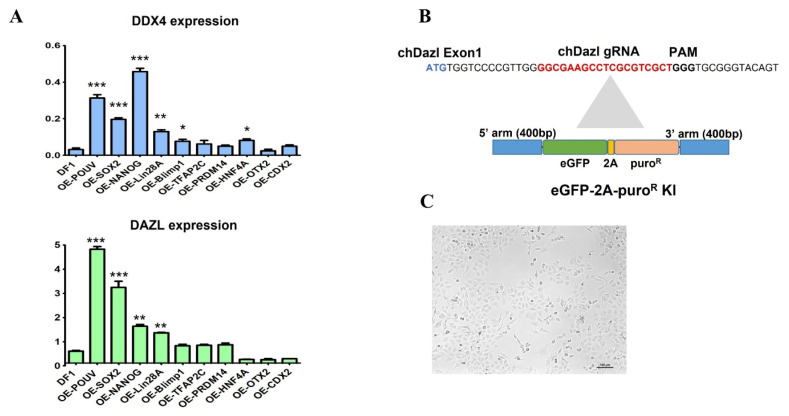
Gene expression analysis in DF1 cells after transfer of each of the 10 selected transcription factor (TF) genes and generation of enhanced green fluorescent protein (eGFP) knock-in (KI) chicken DF1 cells into exon 1 of the *DAZL* gene. (A) Expression profiles of *DDX4* and *DAZL* genes after transfection of each expression vector of the 10 selected transcription factor genes preferentially expressed in chicken germ cells. (B) Schematic diagram of the targeted site in the *DAZL* gene and KI vector with eGFP and puromycin-resistant gene conjugated by 2A sequences. Promoter-less of eGFP-2A-puro^R^ KI vector was inserted into the start codon of the first exon in the chicken *DAZL* gene using a CRISPR/Cas9-mediated system. The expression of eGFP and puro^R^ is controlled by the endogenous chicken *DAZL* promoter specifically in the germ cell lineage. The blue letters are the start codon of the *DAZL* gene. The red and bold letters are guide RNA (gRNA) and protospacer adjacent motif (PAM) sequences, respectively. (C) Morphology of eGFP-2A-puro^R^ KI DF1 cells. Scale: 100 μm. * p<0.05, ** p<0.01, *** p<0.001.

To identify key TFs responsible for maintaining germ cell identity in chicken PGCs, we utilized large-scale gene expression data derived from microarray and WTS analyses. Initially, we examined DEGs in undifferentiated stage X embryos and PGCs compared to somatic cells such as GSCs or CEFs (Supplement 1). Using animalTFDB version 4.0 [23], we matched the upregulated genes in PGCs and ultimately selected 10 TFs associated with pluripotency and germ cell markers by comparing undifferentiated stage X embryos with PGCs (Figure 1). Additionally, we analyzed TF binding sites and identified a set of 10 TFs that had at least one binding site approximately 10 kb upstream of germplasm-associated genes such as *DDX4* and *DAZL*, using the Genomatrix MatInspector software tool. The results revealed that pluripotency markers such as *NANOG* and *POUV* had putative binding sites within the promoter regions of *DDX4* and *DAZL* (Supplement 2), suggesting that the regulation of germ cell fate in chickens operates through a distinct mechanism compared to mammals, involving a unique transcriptional program. To further verify that *DAZL* and *DDX4* gene expression is TF-mediated, we constructed 10 individual gene expression vectors under the control of the CAG promoter and introduced each vector into DF1 cells for overexpression. The results indicated that *DDX4* and *DAZL* expression levels following the overexpression of the selected TFs, including pluripotency markers, were significantly higher than in control DF1 cells (Figure 2A). Among the 10 TFs tested, several TFs significantly upregulated the expression of *DDX4* and *DAZL*, suggesting their involvement in the transcriptional regulation of germplasm genes expression (Figure 2A). Notably, TFs associated with pluripotency showed stronger induction effects, indicating a potential link between pluripotency and germ cell fate regulation.

### Development of CRISPR/Cas9-mediated deleted in azoospermia-like knocked-in enhanced green fluorescent protein DF1 cells

*DAZL* gene expression is a key marker for monitoring germplasm formation and determining the PGC lineage in chickens [6]. To facilitate this, we generated a DAZL-eGFP knocked-in reporter DF1 cell line using a CRISPR/Cas9-mediated knock-in approach. Guide RNA (gRNA) was specifically designed to target the first exon of the chicken *DAZL* gene (Figure 2B), while the homology-directed repair donor template was constructed for a promoter-less eGFP-2A-puroR insertion (Figure 2B). During the Cas9-gRNA-mediated double-strand break repair, the promoter-less eGFP-2A-puroR donor template was occasionally inserted in frame at the beginning of exon 1 in the *DAZL* gene. Subsequently, single-cell-derived DAZL knocked-in eGFP DF1 sublines were established (Figure 2C) and verified through genomic PCR analysis and sequencing using chDAZL KI primers (Supplement 3). Out of 13 single-cell-derived DAZL knocked-in eGFP DF1 sublines analyzed, 9 were confirmed (Supplement 3). The promoter-less eGFP gene was specifically integrated at the transcriptional start site of the *DAZL* gene in DAZL knocked-in eGFP DF1 cells, which were then used in further studies investigating germplasm formation and PGC lineage specification in the chicken model.

### Deleted in azoospermia-like promoter-derived enhanced green fluorescent protein expression after the delivery of 10 transcription factors into chicken DAZL-enhanced green fluorescent protein KI DF1 cells

**Figure 3:**
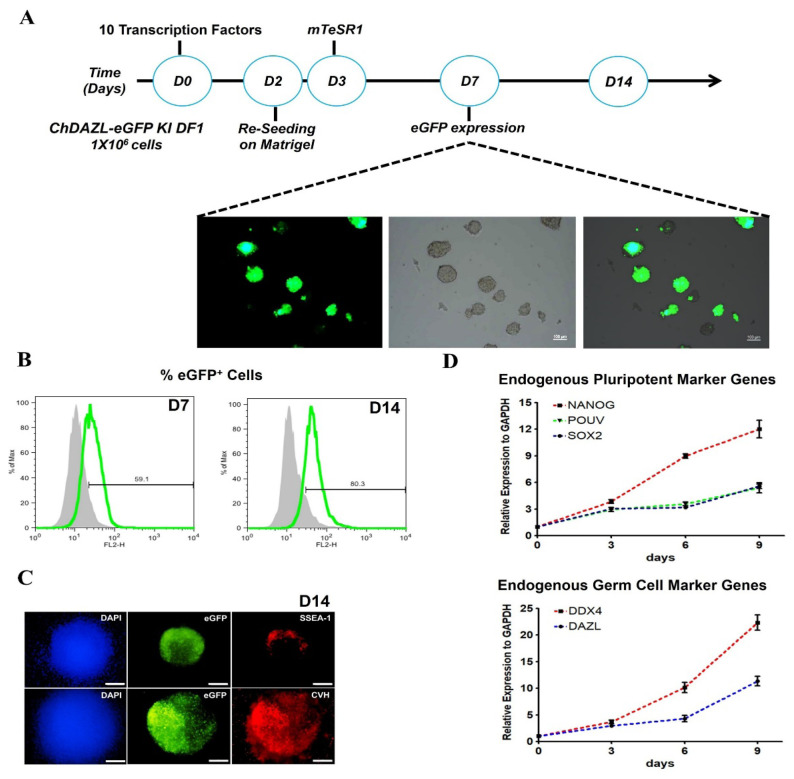
Induction of enhanced green fluorescent protein (eGFP)-expressing germ cells after transfection of 10 transcription factor (TFs) genes. (A) Experimental scheme for induction of germ cells from eGFP-2A-puro^R^ knock-in (KI) DF1 cells through overexpression of 10 TFs. GFP expression was detected 7 days after transfection of the 10 TFs. (B) Flow cytometry analysis of eGFP expression 7 and 14 days after gene delivery of 10 TFs. (C) Immunostaining of the induced germ cell-like cells with germ cell-specific antibodies of SSEA-1 and chicken vasa. (D) Gene expression analysis of endogenous pluripotent and germ cell-specific genes during the induction period.

**Figure 4:**
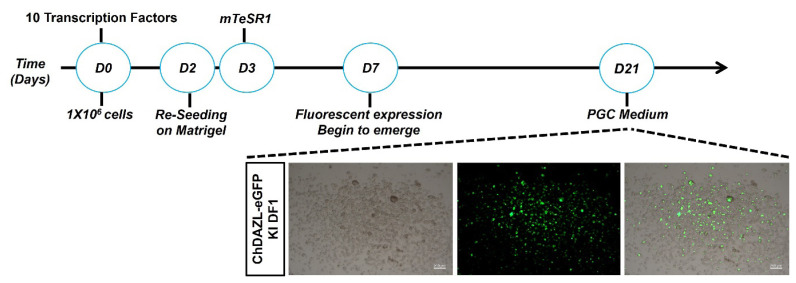
Experimental scheme for induction of germ cells from enhanced green fluorescent protein (eGFP)-2A-puro^R^ knock-in (KI) DF1 cells through overexpression of 10 transcription factors. GFP expression was detected 7 days after transfection of the 10 transcription factors. After 21 days of treatment, the induced germ cell-like cells were maintained with primordial germ cell (PGC) culture media.

We introduced 10 TFs (*NANOG*, *POUV*, SOX2, *LIN28A*, *Blimp1*, *TFAP2C*, *PRDM14*, *HNF4A*, *OTX2* and *CDX2*), selected based on large-scale expression analyses, to transdifferentiate chicken DAZL eGFP knocked-in reporter DF1 cells, resulting in the generation of cells expressing eGFP under the control of the endogenous *DAZL* promoter (Figure 3A). Following transfection with the 10 selected TFs, eGFP expression was observed in chDAZL-eGFP knock-in DF1 cells, indicating activation of the endogenous *DAZL* promoter (Figure 3A). The number of eGFP-positive cells increased over time, particularly at day 14 post-transfection (Figure 3B). Immunofluorescence analysis further confirmed the co-expression of germ cell markers DDX4 and SSEA-1 in eGFP-expressing cells (Figure 3C). qRT-PCR analysis demonstrated that eGFP-positive cells driven by the *DAZL* promoter exhibited an early upregulation of endogenous pluripotency markers, followed by a gradual increase in germplasm-associated genes such as *DDX4* and *DAZL* over time post-transfection (Figure 3D). To promote stable germ cell characteristics, we switched to PGC medium 21 days post-transfection of the 10 TFs, resulting in cells that adopted a single-cell morphology with continuous eGFP expression (Figure 4).

## DISCUSSION

Our findings suggest that the key TFs may be associated with the regulation of germ cell-specific RBPs such as *DDX4* and *DAZL*, by targeting their 5′ upstream promoter regions although direct evidence of transcriptional initiation requires further investigation. Consequently, the germplasm gene expression driven by these key TFs was effective in facilitating *in vitro* gametogenesis in a chicken model.

Germline development differs from somatic tissue development in several key aspects, with germ cell lineage being tightly regulated both spatially and temporally by various molecules, including TFs and RBPs, at the transcriptional and post-transcriptional levels [24,25]. Despite this, the precise mechanisms governing germ cell specification in chickens remain largely unclear. While reconstituting germ cell lineages using pluripotent stem cells has provided a valuable platform for *in vitro* gametogenesis in humans and mice, a similar experimental model is needed for chickens to advance studies in this area.

In this study, we began by investigating the key TFs essential for maintaining germ cell identity in PGCs through large-scale gene expression analysis. While *DAZL* and *DDX4*, well-known for their germ cell-specific expression, have been extensively studied, the mechanisms regulating their expression in PGCs are not yet fully understood. To address this, we analyzed transcriptomes to identify genes significantly expressed in PGCs compared to somatic cells and then cross-referenced them with the AnimalTFDB v4.0 database. Notably, Gene Ontology (GO) analysis of the biological processes associated with DEGs revealed that PGCs are heavily involved in gamete generation, base-excision repair, transcription, and RNA processing, compared to other cell types (Supplement 4). Generally, PGCs exhibit characteristics of both pluripotency and germ cell properties. Among the various significant TFs in PGCs, we focus on evaluating the effect of 10 TFs identified from our analysis, particularly their role in regulating germ cell identity. Recently, several studies have highlighted avian-specific mechanisms underlying germ cell fate determination. In chickens, PGC development is orchestrated by a complex interplay between intrinsic transcriptional regulators and extrinsic signaling cues. Core pluripotency-associated genes such as *NANOG*, *SOX2*, and *POUV* have been shown to play essential roles in maintaining the undifferentiated state of chicken PGCs and ESCs [26]. Among these regulators, *Lin28A* has been identified as a key factor governing both the migratory behavior and maintenance of PGCs [27]. Furthermore, Okuzaki et al [28] demonstrated that *PRDM14* and *BLIMP1* are indispensable TFs required for the proper development and survival of chicken PGCs. Both genes are expressed during early embryogenesis, and their knockdown via *in vivo* RNA interference results in a significant reduction in PGC numbers. In mice, *TFAP2C* and *BLIMP1* are known to play crucial roles in the induction of PGCs during early embryogenesis [29]. In contrast, in chickens, *TFAP2C* has been shown to interact with RNA polymerase II through a long intergenic non-coding RNA (lincRNA), functioning as an epigenetic regulator by targeting the active histone mark H3K4me3, thereby promoting *CDX2* expression in ESCs [30]. Moreover, Zhang et al [31] identified *OTX2* as a key TF involved in the segregation of germline and somatic lineages. In this context, *HNF4A* has also emerged as a pivotal regulator, serving as a hub gene essential for maintaining germ cell identity in chicken PGCs.

Next, we investigated whether the 10 selected TFs with putative binding sites in the promoter regions of germplasm genes can directly regulate chicken *DDX4* and *DAZL*. The expression of germ cell-specific RBPs is crucial for PGC specification. At the post-transcriptional level, RBPs regulate RNA localization and translation, playing an essential role in PGC lineage development. Observation of CVH-containing cytoplasmic structures suggests that the chicken germline is determined by maternally inherited factors in the germ plasm [6]. Several studies have shown that DDX4 plays a critical role in the formation of the germplasm and gametogenesis [4]. More importantly, the DAZL protein, which belongs to the RRM DAZ family, contains a DAZ-like domain and an RRM (RNA recognition motif) domain. It is likely an RBP that plays a central role during gametogenesis by binding to the 3′-UTR of mRNA, thereby regulating the translation of key transcripts [32]. Overall, we found that the regulation of chicken germ cell fate involves distinct mechanisms compared to that of mammals, displaying a unique transcriptional program. These results show that a set of TFs related to pluripotent markers directly impacts the expression of RBPs such as *DDX4* and *DAZL* to maintain the germ cell characteristics. Furthermore, we established a precise reporter system to trace germplasm formation and chicken PGC lineage specification. After introducing the 10 TFs into chDAZL-eGFP KI DF1 cells, we confirmed the specification of eGFP expression driven by the DAZL promoter. Cells expressing eGFP exhibited characteristics of germ cells, such as DDX4 and SSEA-1 expression. These cells also showed a significant increase in endogenous pluripotent markers (*NANOG*, *SOX2* and *POUV*) and a gradual increase in endogenous germplasm markers (*DDX4* and *DAZL*) after transfection (Figure 3). Collectively, our integrated approach was effective for evaluating selected TFs with putative binding sites in the DAZL promoter. Furthermore, switching PGC medium at D21 post-transfection of TFs (Figure 4) indicated that cell morphology changed to a single cell type and eGFP was continuously expressed.

At this stage, the generation of germ cells remains incomplete, further requiring specific conditions to effectively induce primordial germ cells (iPGCs) such as precise modulation of signalling pathways and epigenetic states. Additionally, the use of DF-1 cells as the parental line imposes inherent limitations, as they do not possess the native epigenetic landscape. This may restrict the full reprogramming potential and highlights the need for intact cells such CEFs in future studies.

Taken together, our findings offer valuable insights into chicken germ cells through screening to determine the key TFs involved in the induction of *DAZL* gene expression, highlighting the role of key TFs and RBPs. These results hold great promise for avian research and pave the way for *in vitro* germ cell induction, with potential applications of *in vitro* models for studying germ cell-specific gene regulatory pathways in avian species. Such systems may serve as powerful platforms for generating germline-competent cells, modeling the developmental trajectory of avian germ cells, preserving endangered genetic resources, and facilitating targeted genome engineering in poultry species.

## CONCLUSION

Chicken DAZL knock-in reporter DF1 cells offer a robust *in vitro* system for monitoring DAZL gene activation and tracking the induction of iPGCs. This reporter system is expected to be a valuable tool for exploring the regulatory pathways and mechanisms governing pluripotency and germ cell fate in a chicken model.

## Figures and Tables

**Table 1:** Primers used for this study for the gene expression analysis

No.	Gene symbol	Forward	Reverse	Product size (bp)
1	*GAPDH*	CCTCTCTGGCAAAGTCCAAG	CATCTGCCCATTTGATGTTG	200
2	*DDX4*	GCTATGGAGGAGGACTGGGA	CTCTCTGTACAGCCCTTGCC	311
3	*DAZL*	CCATTCGTCAACAACCTGCC	TCCAGGAAAATCTCTTTTGTTTGT	256

**Table 2:** Primers used for this study for expression of endogenous pluripotency markers and germplasm markers

No.	Gene symbol	Forward	Reverse	Product size (bp)
1	Endo *NANOG*	CTACCAATGT GGATT ATGAC	TTGCAAAGGGTTAGCTATGC	205
2	Endo *POUV*	AGCAGCCACTGAACAGCAGC	CAGGAGGGGCTGACCCCACA	110
3	Endo *SOX2*	GAGGGCTCCTTGCCAAGC	TTTTATTAGAAATTCTGGACCTTT	248
4	Endo *DDX4*	TGTTGCTTCACTTGGTGCCC	AAGTGTGTTTACAAAGCATCA	241
5	Endo *DAZL*	GTCTGAGGAACACCTTTGTA	TCTTCAACTCTTACAACTTCA	200

## SUPPLEMENTARY MATERIAL

**Supplement 1.** Scatter plot revealing the upregulation of differentially expressed genes (DEGs) in undifferentiated stage X embryos and PGCs compared to somatic cells such as CEFs.

**Supplement 2.** The predicted binding sites of *NANOG, POUV* and *Sox2* in the promoter of the *DDX4* or *DAZL* genes.

**Supplement 3.** Generation of eGFP knock-in (KI) chicken DF1 cells into exon 1 of the DAZL gene.

**Supplement 4.** The biological process terms of gene ontology (GO) on genes that were significantly expressed in PGC.
