# Supplementary material for: Induction of germ cell-like cells from deleted in azoospermia-like enhanced green fluorescent protein gene knock-in chicken somatic cells via transgenic expression of pluripotency and germ cell-specific transcription factors
Source: Anim Biosci. 2025 Aug 12;39(1):250233. doi: 10.5713/ab.25.0233 (PMC12754494; doi:10.5713/ab.25.0233)
Supplement: Supplementary file 1 [file ab-25-0233-Supplementary-1.pdf]

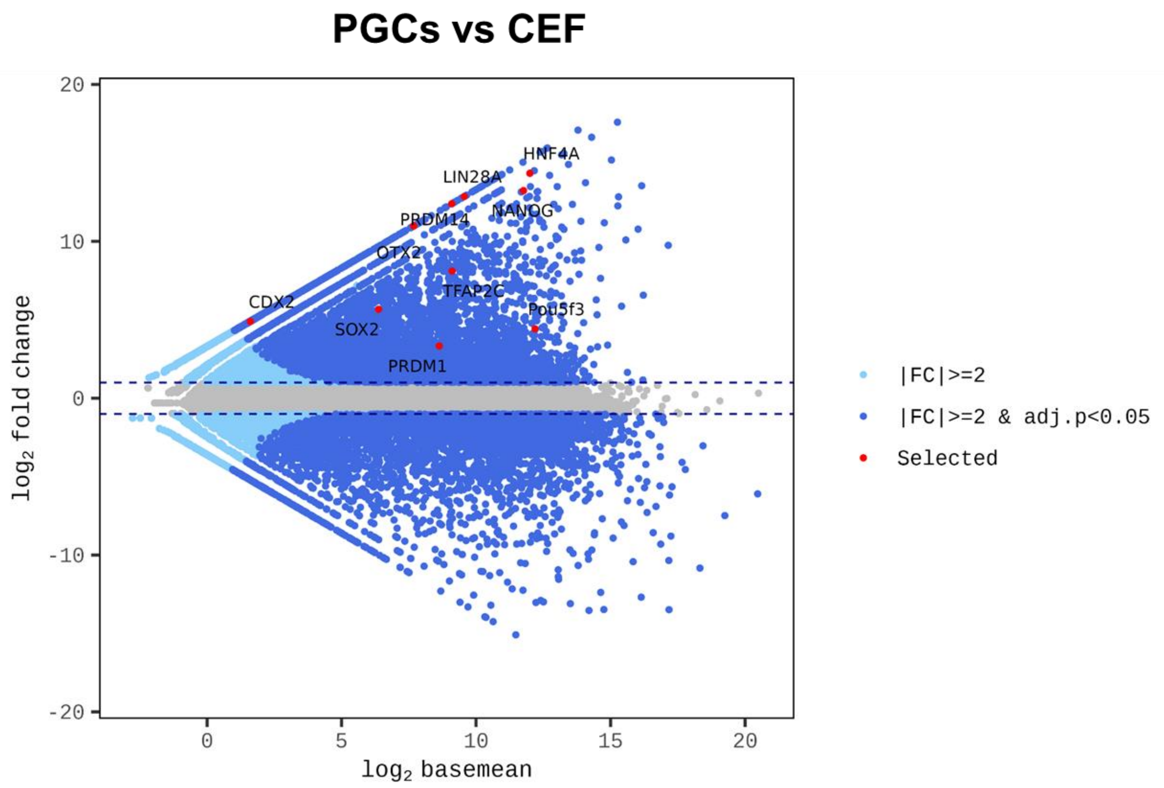

Supplement 1. Scatter plot revealing the upregulation of differentially expressed genes (DEGs) in undifferentiated stage X embryos and PGCs compared to somatic cells such as CEFs.
