# Supplementary material for: Induction of germ cell-like cells from deleted in azoospermia-like enhanced green fluorescent protein gene knock-in chicken somatic cells via transgenic expression of pluripotency and germ cell-specific transcription factors
Source: Anim Biosci. 2025 Aug 12;39(1):250233. doi: 10.5713/ab.25.0233 (PMC12754494; doi:10.5713/ab.25.0233)
Supplement: Supplementary file 2 [file ab-25-0233-Supplementary-2.pdf]

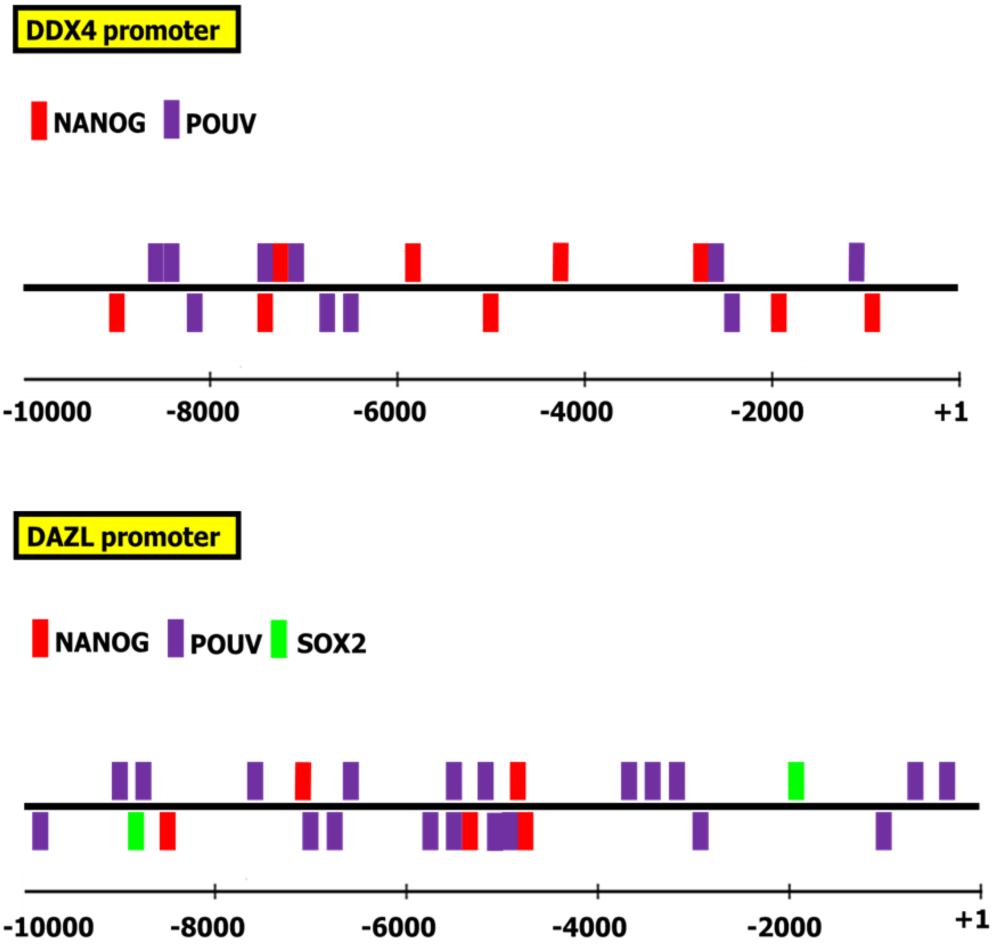

Supplement 2. The predicted binding sites of *NANOG*, *POUV* and *Sox2* in the promoter of the *DDX4* or *DAZL* genes.
