# Supplementary material for: Induction of germ cell-like cells from deleted in azoospermia-like enhanced green fluorescent protein gene knock-in chicken somatic cells via transgenic expression of pluripotency and germ cell-specific transcription factors
Source: Anim Biosci. 2025 Aug 12;39(1):250233. doi: 10.5713/ab.25.0233 (PMC12754494; doi:10.5713/ab.25.0233)
Supplement: Supplementary file 3 [file ab-25-0233-Supplementary-3.pdf]

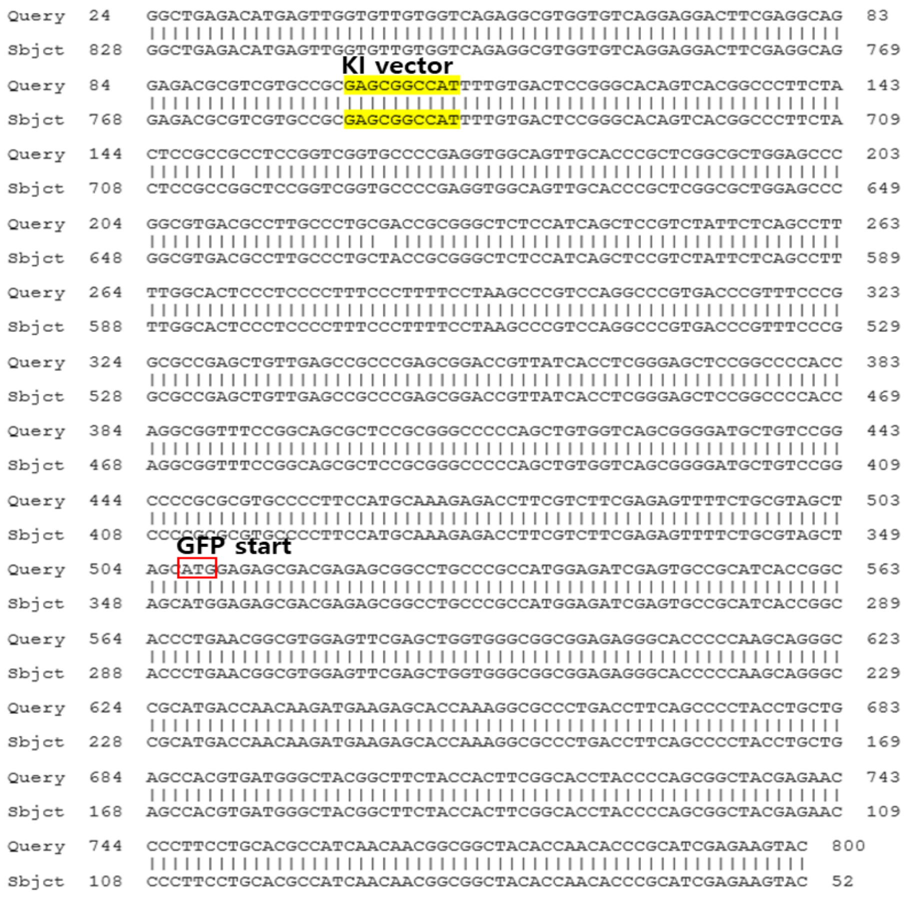

DF1 Dazl-GFP KI#4 target sequencing

Supplement 3. Generation of eGFP knock-in (KI) chicken DF1 cells into exon 1 of the DAZL gene. Sequencing analysis of the targeted site in the chicken *DAZL* gene after generation of eGFP-2A-puro^R KI DF1 cells.
